# Supplementary material for: Development of Pollen Parent Cultivar-Specific SCAR Markers and a Multiplex SCAR-PCR System for Discrimination between Pollen Parent and Seed Parent in Citrus
Source: Plants (Basel). 2023 Nov 27;12(23):3988. doi: 10.3390/plants12233988 (PMC10708447; doi:10.3390/plants12233988)
Supplement: Supplementary file 1 [file plants-12-03988-s001.zip › plants-2722245-supplementary/plants-2722245-proofed supplementary/Supplementary +Figure+S1.pdf]

Figure S1. DNA sequence of 'Asumi' cultivar-specific RAPD PCR amplification product.

1 CTCAGCCCAG AACCCGAACC AATGGCTCAA AATAACAACC AAACACTTAA

51 AGAGTTGGCA ACTCCTAACT TGGACCAGCA ACCCTTATGC ATTGAGAATC

101 CAAACCCTCA GGTAAACTTT GAACTCAAAT CTGGGATGAT TCATCTTCTT

151 CATATCTGCA ATGCTTTGCA ATCCATTGAC GTTTCTTTTT GTAAAAGGGT

201 AATGGAGCTG GTGGAGGGGG AGGATCTTGC GGTGGTTTGG GGTCACAATG

251 GTGACATAAG ATTGGAGATT TTTTCTTTTT GTTTTTCTTT TTCCTTGTGG

301 TGGCATAATC ATTATCCTCT TCCTAATCAT CCCAGCAGGG ACAATTAGGG

351 TCACACATGC CAGAGCCAGG GGCATCCCAT AGGAAATGGC CAATTTACTT

401 GGCTGGATAA ACTGGGTAAC CTTCAGAGTT GAACCCTGTA ATGGGTAGAT

451 CTTCCTGGGC TGAG
