# Supplementary material for: Development of Pollen Parent Cultivar-Specific SCAR Markers and a Multiplex SCAR-PCR System for Discrimination between Pollen Parent and Seed Parent in Citrus
Source: Plants (Basel). 2023 Nov 27;12(23):3988. doi: 10.3390/plants12233988 (PMC10708447; doi:10.3390/plants12233988)
Supplement: Supplementary file 1 [file plants-12-03988-s001.zip › plants-2722245-supplementary/plants-2722245-proofed supplementary/Supplementary+Figure+S2.pdf]

Figure S2. Identical sequence amplified in two varieties for the production of control markers for discrimination of ‘Asumi’ and ‘Asuki’ varieties.

1 TGAGTCCAAA CCGGAAGTAG GTGGAAAACT TCGGTCAATC AGCCCCGCTC

51 TCCCCAACCT GCTCTTCTTT AACACATCAC TACCCCTTTG TACACCTCGA

101 AGCCACTCCA CAAACTTTTT CACCTCTTTC CTCACCTCCT AAACTGGGGA

151 TGATCCTTTA GCACACGTCA CACCTGCAAA ATCATGGAGA ACTCAAGGTT

201 AGCGAGACAT TTACCCGAAG GATTACAATA AAGAACAGTA AATAAGGGAG

251 ATACTTACAG TCTACATTGA AGGAATTTAG GATCTCCACA TGGTTGCCCT

301 TATCGCCTAG AGTCTCTATC CCCCACTTAC CTTCAGTAAC AAACCACCCA

351 TTTTTGTAAC CCTTCTTCAA GGCGGGGTCC CCTTTAACAA TATTGCCATG

401 ATCACCCCAA GCAGCGAAGT AAACAATAAC GAGACCCTTG AGATTTTTGA

451 ATTGGTAAAG GTCCCTCAAT TTTCAAATAG AAATATCACG CCTCAATACC

501 TCCGACCATA GCACCTGCAA GCCCGTAAGT GTCCTCCACC CTAGAAGGGA

551 CAGCTGCCCA TGGGCCACCC CTAAATAACT AAACATCTTC ATATAGAAAG

601 GTTGGAGGGG CAAGCAAAGT TCAAGATCAA AGAGGTCAAT GTGAAGAGCA

651 ATCCCGTCAG ATTTAGGGCA CTCAGGTCGT TCATCCACAT GGGGTACCTT

701 AAGCTGGACA GAAGGAGGAA TATTGTATTT GGTTCTAATT CGTACGCAGT

751 C
