# Supplementary material for: Development of Pollen Parent Cultivar-Specific SCAR Markers and a Multiplex SCAR-PCR System for Discrimination between Pollen Parent and Seed Parent in Citrus
Source: Plants (Basel). 2023 Nov 27;12(23):3988. doi: 10.3390/plants12233988 (PMC10708447; doi:10.3390/plants12233988)
Supplement: Supplementary file 1 [file plants-12-03988-s001.zip › plants-2722245-supplementary/plants-2722245-proofed supplementary/Supplementary+Table+S1.pdf]

Table S1. Primer sets used for SCAR marker development.

Primer name	Primer sequence (5'–3')	(bp)	Location of primers
UBC 218F	CTCAGCCCAGAACCCGAACCAATGGCTC	28	1
UBC F1	CAGCAACCCTTATGCATTGA	20	76
UBC F2	CCT AAC TTG GAC CAG CAA CC	20	64
UBC F3	ACT TGG ACC AGC AAC CCT TA	20	68
UBC F1-1	CTCAGCCCAGAACCCGAACCAATGGCTC	28	1
UBC F2-1	GAGTTGGCAACTCCTAACTTGGACCAGC	28	51
UBC F3-1	GTAATGGAGCTGGTGGAGGGGGAGGAT CTTG	31	198
UBC 218R	CAGGAAGATCTACCCATTACAGGGTTCAAC	30	457
UBC R1	GCCACCACAAGGAAAAAGAA	20	304
UBC R2	ATGCCACCACAAGGAAAAAG	20	306
UBC R3	TGATTATGCCACCACAAGGA	20	311
UBC R1-1	CAGGAAGATCTACCCATTACAGGGTTCAAC	30	457
UBC R2-1	CTGGCATGTGTGACCCTAATTGTCCCTGCTG	31	373
UBC R3-1	GCATGTGTGACCCTAATTGTCCCTGCTG	28	370
SRAPF1	GAGTCCAAACCGGAAGTAGG	20	2
SRAPF2	GGTCAATCAGCCCCGCTCTCC	21	32
SRAPF3	CAAACCGGAAGTAGGTGGAAAA	22	7
SRAPF4	CTGGGGATGATCCTTTAATC	20	144
SRAPF5	CGAGACATTTACCCGAAGGA	20	203
SRAPR1	GACTGCGTACGAATTAGAAC	20	732
SRAPR2	TCCTTCGGGTAAATGTCTCG	20	203
SRAPR3	GATTTTGCAGGTGTGACGTG	20	164
SRAPR4	ATAAGGGCAACCATGTGGAG	20	284
SRAPR5	CGCCTTGAAGAAGGGTTACA	20	355
