# Supplementary material for: Development of Pollen Parent Cultivar-Specific SCAR Markers and a Multiplex SCAR-PCR System for Discrimination between Pollen Parent and Seed Parent in Citrus
Source: Plants (Basel). 2023 Nov 27;12(23):3988. doi: 10.3390/plants12233988 (PMC10708447; doi:10.3390/plants12233988)
Supplement: Supplementary file 1 [file plants-12-03988-s001.zip › plants-2722245-supplementary/plants-2722245-proofed supplementary/Supplementary+Table+S2.pdf]

Table S2. RAPD-SCAR markers used for 'Asumi' and 'Asuki' cultivar selection.

No.	SCAR primer name	Primer sequence (5'–3')	Size of amplified product (bp)
1	UBC 218F<br>UBC 218R	CTCAGCCCAGAACCCGAACCAATGGCTC<br>CAGGAAGATCTACCCATTACAGGGTTCAAC	1–457
2	UBC F1<br>UBCR3	CAGCAACCCTTATGCATTGA<br>TGATTATGCCACCACAAGGA	76–311
3	UBC F1<br>UBC R1-1	CAGCAACCCTTATGCATTGA<br>CAGGAAGATCTACCCATTACAGGGTTCAAC	76–457
4	UBC F1<br>UBC R2-1	CAGCAACCCTTATGCATTGA<br>CTGGCATGTGTGACCCTAATTGTCCCTGCTG	76–373
5	UBC F1<br>UBC R3-1	CAGCAACCCTTATGCATTGA<br>GCATGTGTGACCCTAATTGTCCCTGCTG	76–370
6	UBC F2<br>UBC R3	CCTAACTTGGACCAGCAACC<br>TGATTATGCCACCACAAGGA	64–311
7	UBC F2<br>ubcR1-1	CCTAACTTGGACCAGCAACC<br>CAGGAAGATCTACCCATTACAGGGTTCAAC	64–457
8	UBC F2<br>ubcR3-1	CCTAACTTGGACCAGCAACC<br>GCATGTGTGACCCTAATTGTCCCTGCTG	64–370
9	UBC F3<br>UBC R1-1	ACTTGGACCAGCAACCCTTA<br>CAGGAAGATCTACCCATTACAGGGTTCAAC	68–457
10	UBC F3<br>UBC R2-1	ACTTGGACCAGCAACCCTTA<br>CTGGCATGTGTGACCCTAATTGTCCCTGCTG	68–373
11	UBC F3<br>UBC R3-1	ACTTGGACCAGCAACCCTTA<br>GCATGTGTGACCCTAATTGTCCCTGCTG	68–370
12	UBC F1-1<br>UBC R3	CTCAGCCCAGAACCCGAACCAATGGCTC<br>TGATTATGCCACCACAAGGA	1–311
13	UBC F1-1<br>UBC R1-1	CTCAGCCCAGAACCCGAACCAATGGCTC<br>CAGGAAGATCTACCCATTACAGGGTTCAAC	1–457
14	UBC F1-1<br>UBCR2-1	CTCAGCCCAGAACCCGAACCAATGGCTC<br>CTGGCATGTGTGACCCTAATTGTCCCTGCTG	1–373
15	UBC F1-1<br>UBCR3-1	CTCAGCCCAGAACCCGAACCAATGGCTC<br>GCATGTGTGACCCTAATTGTCCCTGCTG	1–370
16	UBC F2-1<br>UBCR1	GAGTTGGCAACTCCTAACTTGGACCAGC<br>GCCACCACAAGGAAAAAGAA	51–304
