# Supplementary material for: Development of Pollen Parent Cultivar-Specific SCAR Markers and a Multiplex SCAR-PCR System for Discrimination between Pollen Parent and Seed Parent in Citrus
Source: Plants (Basel). 2023 Nov 27;12(23):3988. doi: 10.3390/plants12233988 (PMC10708447; doi:10.3390/plants12233988)
Supplement: Supplementary file 1 [file plants-12-03988-s001.zip › plants-2722245-supplementary/plants-2722245-proofed supplementary/Supplementary+Table+S3.pdf]

Table S3. SRAP-SCAR markers used for control discrimination.

No.	SRAP-SCAR primer name	Primer sequence (5'–3')	Size of amplified product (bp)
1	SRAPF2	GGTCAATCAGCCCCGCTCTCC	32–203
	SRAPR2	TCCTTCGGGTAAATGTCTCG	
2	SRAPF2	GGTCAATCAGCCCCGCTCTCC	32–164
	SRAPR3	GATTTTGCAGGTGTGACGTG	
3	SRAPF2	GGTCAATCAGCCCCGCTCTCC	32–284
	SRAPR4	ATAAGGGCAACCATGTGGAG	
4	SRAPF2	GGTCAATCAGCCCCGCTCTCC	32–355
	SRAPR5	CGCCTTGAAGAAGGGTTACA	
5	SRAPF3	CAAACCGGAAGTAGGTGGAAAA	7–203
	SRAPR2	TCCTTCGGGTAAATGTCTCG	
6	SRAPF3	CAAA CCGGAAGTAG GTGGAAAA	7–284
	SRAPR4	ATAAGGGCAACCATGTGGAG	
7	SRAPF5	CGAGACATTTACCCGAAGGA	203–284
	SRAPR4	ATAAGGGCAACCATGTGGAG	
8	SRAPF5	CGAGACATTTACCCGAAGGA	203–355
	SRAPR5	CGCCTTGAAGAAGGGTTACA	
