# Supplementary material for: Development of Pollen Parent Cultivar-Specific SCAR Markers and a Multiplex SCAR-PCR System for Discrimination between Pollen Parent and Seed Parent in Citrus
Source: Plants (Basel). 2023 Nov 27;12(23):3988. doi: 10.3390/plants12233988 (PMC10708447; doi:10.3390/plants12233988)
Supplement: Supplementary file 1 [file plants-12-03988-s001.zip › plants-2722245-supplementary/plants-2722245-proofed supplementary/Supplementary+Table+S4.pdf]

Table S4. Citrus cultivars used for the application of the selected SCAR markers.

No.	Cultivar name	Parentage or scientific name
S1	‘Asumi’	‘Okisu 46 gou’ × ‘Harumi’
S2	‘Asuki’	′Okisu 46 gou’ × ‘Harumi’
S3	‘Okitsu wase’	*C. unshiu* Marc.
S4	‘Miyagawa wase’	*C. unshiu* Marc.
S5	‘Nichinan 1gou’	*C. unshiu* Marc.
6S	‘Haryejosaeng’	*C. unshiu* Marc.
S7	‘Kiyomi’	*C.* hybrid (‘Miyagawa wase’ × ‘Trovita’ orange)
S8	‘Wilking’	*C. reticulata*
S9	‘Nova’	*C.* hybrid (‘Clementine’ × ‘Orlando’)
S10	‘Lee’	*C. reticulata*
S11	‘Encore’	*C. reticulata*
S12	‘Ootaponkan’	*C. reticulata*
S13	‘Hayaka Ponkan’	*C. reticulata*
S14	‘Murcott’	*C. reticulata*
S15	‘Harumi’	*C.* hybrid (‘Kiyomi’ × ‘Ponkan F2432’)
S16	‘Shiranuhi’	‘Kiyomi’ × ‘Nakno 3 gou’ ponkan
S17	‘Kanpei’	‘Nishinokaori’ × Ponkan
S18	‘Setoka’	*C.* hybrid (‘Kiyomi’ × ‘Encore’) × ‘Murcott’
S19	‘Haruka’	*C.* hybrid (natural crossing seedling of ‘Hyuganatsu’)
S20	‘Winter Prince’	*C.* hybrid (‘Harehime’ × ‘Ootaponkan’)
S21	‘Tamnaneunbong’	*C.* hybrid (nucellar seedling of ‘Shiranuhi’)
S22	‘Yellow ball’	*C. reticulata* (‘Kiyomi’ × ‘Haruka’)
S23	‘Minihyang’	*C. reticulata* (‘Kishu mikan’ × ‘Ootaponkan’)
S24	‘Tsunokaori’	*C.* hybrid (‘Kiyomi’ × ‘Okitsu wase’)
S25	‘Sinyegam’	*C.* hybrid (‘Kiyomi’ × ‘Wilking’)
S26	‘Mihaya’	*C.* hybrid
S27	‘Miraehyang’	*C.* hybrid (‘Eime Kashi 28 gou’ × ‘Hayaka Ponkan’)
S28	‘Eime Kashi 28 gou’	*C.* hybrid (‘Nankou’ × ‘Amakusa’)
S29	‘Pungkwang navel’	*C. sinensis* (nucellar seedling of ‘Washington navel’ × *C. Platymamma* ‘Byungkyul’)
S30	‘Washingon navel’	*C. sinensis*
