# Supplementary material for: Development of Pollen Parent Cultivar-Specific SCAR Markers and a Multiplex SCAR-PCR System for Discrimination between Pollen Parent and Seed Parent in Citrus
Source: Plants (Basel). 2023 Nov 27;12(23):3988. doi: 10.3390/plants12233988 (PMC10708447; doi:10.3390/plants12233988)
Supplement: Supplementary file 1 [file plants-12-03988-s001.zip › plants-2722245-supplementary/plants-2722245-proofed supplementary/Supplementary+Table+S5.pdf]

Table S5. Mandarin cultivars used for the application of the selected SCAR markers.

No.	Cultivar name	Scientific name
1	'Dancy'	*C. reticulata*
2	'Natsumi'	*C. reticulata*
3	'Byungkyul'	*C. platymamma*
4	'Jinkyul'	*C. sunki*
5	'Binkyul'	*C. leiocarpa*
6	'Southern Yellow'	*C.* hybrid
7	'King'	*C. nobilis* or *C. reticulata*
8	'Kinnow'	*C. reticulata*
9	'Dongjeonkyul'	*C. erythrosa*
10	'Fortune'	*C. reticulata*
11	'Page'	*C. reticulata* ('Minneola' × 'Clementine')
12	'Kinokuni'	*C. kinokuni*
13	'Clementine'	*C. clementina*
